# Approaching soft X-ray wavelengths in nanomagnet-based microwave technology

**DOI:** 10.1038/ncomms11255

**Published:** 2016-04-11

**Authors:** Haiming Yu, O. d' Allivy Kelly, V. Cros, R. Bernard, P. Bortolotti, A. Anane, F. Brandl, F. Heimbach, D. Grundler

**Affiliations:** 1 Physik Department E10, Technische Universität München, James-Franck-Strasse 1, D-85748 Garching bei München, Germany; 2 Fert Beijing Institute, School of Electronic and Information Engineering, Beihang University, Xueyuan Road 37, Beijing 100191, China; 3 Unité Mixte de Physique, CNRS, Thales, Univ Paris-Sud, Université Paris-Saclay, 91767 Palaiseau, France; 4 Laboratory of Nanoscale Magnetic Materials and Magnonics, Institute of Materials, School of Engineering, École Polytechnique Fédérale de Lausanne, STI-IMX-LMGN, Station 17, CH-1015 Lausanne, Switzerland

## Abstract

Seven decades after the discovery of collective spin excitations in microwave-irradiated ferromagnets, there has been a rebirth of magnonics. However, magnetic nanodevices will enable smart GHz-to-THz devices at low power consumption only, if such spin waves (magnons) are generated and manipulated on the sub-100 nm scale. Here we show how magnons with a wavelength of a few 10 nm are exploited by combining the functionality of insulating yttrium iron garnet and nanodisks from different ferromagnets. We demonstrate magnonic devices at wavelengths of 88 nm written/read by conventional coplanar waveguides. Our microwave-to-magnon transducers are reconfigurable and thereby provide additional functionalities. The results pave the way for a multi-functional GHz technology with unprecedented miniaturization exploiting nanoscale wavelengths that are otherwise relevant for soft X-rays. Nanomagnonics integrated with broadband microwave circuitry offer applications that are wide ranging, from nanoscale microwave components to nonlinear data processing, image reconstruction and wave-based logic.

## Abstract (web summary)

Integrating spin waves with microwave circuits is promising for microwave nanoelectronics. Here, Yu *et al*. demonstrate a reconfigurable microwave-to-magnon transducer by covering yttrium iron garnet with small arrays of tailored magnetic nanodisks, which transmits microwave signals via sub-100-nanometer wavelength spin waves.

## Graphical abstract

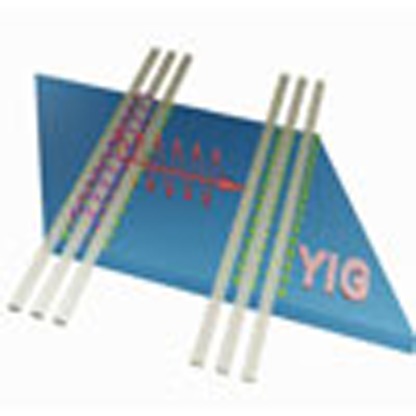

Magnonics is an emerging technology for low-power signal transmission and data processing based on spin waves (magnons) propagating in magnetic materials^1^,^2^,^3^,^4^,^5^. In contrast to, for example, plasmons in metals^6^, magnons exist also in insulators, overcoming the damping issue. Thanks to the rapid advances of nanotechnology, magnonics is currently seeing an outburst. The wavelength 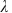 of spin waves processed by magnonic crystals and magnonic waveguides have diminished from the millimetre to micrometre length scale at a fast pace^7^,^8^,^9^. Evidencing a wavelength of 900 nm, Demidov *et al*.^10^ demonstrated a powerful wavelength-conversion technique, removing restrictions imposed by the size of microwave antennas. Still, for the real breakthrough, spin waves should be generated and manipulated down to the few 10-nm length scale. At this scale, exchange interaction dominates enabling large group velocities and fast signal transmission. From such spin waves, nanoscale microwave devices operating from GHz to THz can be conceived, for future mobile communications^11^ and efficient computing based on coherent data processing^12^. However, sub-100 nm magnonics was argued to be far from realization^4^. Inelastic light scattering is widely used for studying propagating spin waves with 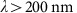
13,14. Neutron scattering^15^, electron loss spectroscopy^16^ and scanning tunnelling microscopes^17^ have addressed smaller 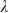. Unfortunately, none of these techniques allow direct integration on a chip. In the past, on-chip spin-wave emitters were realized by parametric pumping^18^ and spin-transfer torque nanopillars^13^,^14^. However, the extremely large microwave signal and complex spin-wave emission, respectively, make the low-power consumption and coherent processing of information in interconnected magnonic networks unrealistic^12^. Au *et al*.^19^ proposed a resonant microwave-to-magnon transducer exploiting spin-precessional motion in a ferromagnetic nanowire placed on top of a magnonic conduit. Following this intriguing concept, they evidenced spin-wave excitation at 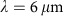. Our approach tackles the long-standing problem how to integrate sub-100 nm magnonics on-chip with microwave circuits^4^.

In this paper, we report the inductive generation and detection of plane-wave spin waves in the low-power linear regime with a wavelength 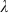 of 88±2 nm. This record value of 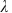 is achieved in thin yttrium iron garnet (YIG) by using conventional coplanar waveguides (CPWs) that cover small arrays of tailored magnetic nanodisks. The nanodisks reside between the CPWs and YIG, and are operated as microwave-to-magnon transducers^19^. For them, we use materials established in spintronics and magnonics, that is, polycrystalline Ni_80_Fe_20_ (Py) and amorphous Co_20_Fe_60_B_20_ (CoFeB). Spin waves with wave vectors of up to 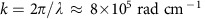 show large signal-to-noise ratios and are exchange dominated exhibiting enlarged group velocities. At the same time, we discover our microwave-to-magnon transducers to be reconfigurable. They provide resonantly enhanced spin-wave amplitudes that are tuned ON and OFF by writing saturated and unsaturated states, respectively, on the disks. Our results pave the way for interconnecting conventional microwave electronics with exchange-dominated spin waves and substantially advancing magnonics-based technologies.

## Results

### Enhanced magnon excitation via resonantly driven nanodisks

**Figure 1:**
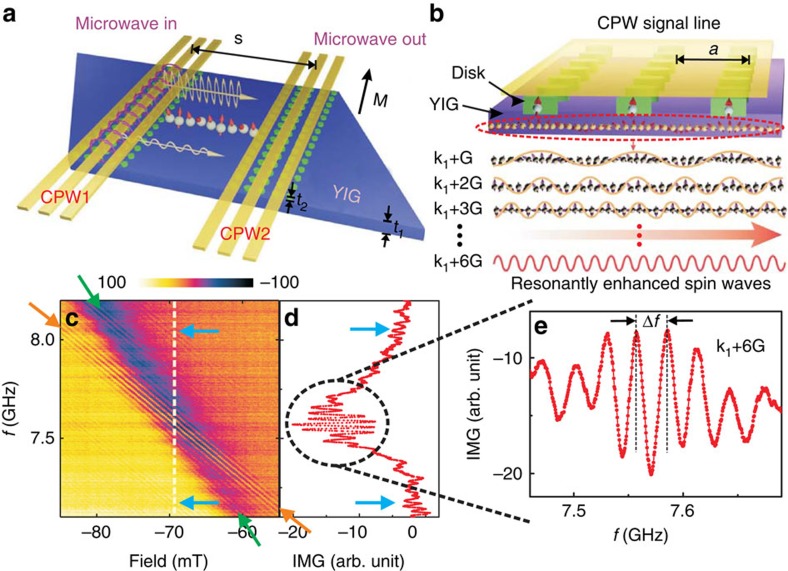
Resonantly driven nanodisks for injection and detection of large-amplitude spin waves in thin YIG. (**a**) Sketch of the experiment. Large-magnetization ferromagnetic nanodisk arrays (green) were positioned between CPWs (yellow) and insulating YIG (violet). The magnetization *M* of YIG is parallel to the CPWs. (**b**) Sketched microwave-to-magnon transduction: the resonant spin-precessional motion in disks (large arrows) is exploited to excite short-wavelength spin waves (small arrows highlighted by the oval) in YIG. Depending on the frequency, different wave vectors *k*_1_+*nG* (*n*=1, 2, 3, …) are induced. 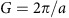 denotes a reciprocal lattice vector perpendicular to the CPW. Spin waves propagate to the detector CPW indicated by the horizontal arrow. (**c**) Colour-coded transmission signal *S*_12_ monitoring spin-wave propagation between CPW1 to CPW2. Green and orange arrows guide the eyes and highlight two branches that cross near the centre of the graph. Green (orange) arrows indicate the resonant excitation of the permalloy nanodisks (YIG at large wave vector *k*_SW_). (**d**) Transmission spectrum *S*_12_ at −69 mT (broken line in **c**) displaying propagation of spin waves through YIG with enlarged amplitude when permalloy nanodisks resonate together with YIG. Blue arrows indicate non-resonant spin-wave excitation. (**e**) Enlarged oscillating transmission signal (imagninary (IMG) part) around 7.6 GHz attributed to *k*_SW_=*k*_1_+6*G*=48 rad μm^−1^=4.8 × 10^5^ rad cm^−1^. The corresponding wavelength amounts to 131±3 nm.

Figure 1a,b illustrates sample and experiment. CPWs were integrated to ferrimagnetic YIG with a thickness *t*_1_=20 nm. Underneath the CPWs, nanodisks of thickness *t*_2_=15 nm were fabricated from either evaporated Py or magnetron sputtered CoFeB using electron beam lithography and lift-off processing (Supplementary Fig. 1). The nanodisks with diameter of 350±5 nm were arranged on a square lattice with a lattice constant *a*=800 nm. The real and imaginary part of scattering parameters *S*_*pq*_ were measured in reflection and transmission configuration using a vector network analyser (VNA) connected to the CPWs (*p*=1,2 and *q*=1, 2 denote the different ports of the VNA and the CPWs). In this work we show the imaginary part of *S*_12_ and not the modulus to benefit from the phase resolution of the VNA and extract the group velocity *v*_g_ from oscillating transmission signals^20^,^21^ (Methods). The microwave magnetic field *h*_rf_ generated by a CPW was inhomogeneous and provided excitation in the linear regime at a distinct set of wave vectors **k**_i_ with *i*=1, 2, 3, … as explored in ref. 20. The propagation distance *s* between CPWs was 30 μm.

We found that the resonant spin-precessional motion, that is, ferromagnetic resonance (FMR), of saturated nanodisks occurred at much larger frequency *f*_0_ compared with the uniform mode of the YIG film with wave vector *k*_SW_=0. Only for spin waves in YIG with large *k*_SW_, the nanodisks were in resonance with spin precession in YIG. In Fig. 1c, we show colour-coded transmission data *S*_12_ obtained on the Py/YIG hybrid sample. We focus on a field regime where field-dependent FMR of Py nanodisks (branch highlighted by green arrows) becomes degenerate with spin waves in YIG (branch indicated by orange arrows). In Fig. 1d,e, the line spectrum *S*_12_ taken at the crossing point at *μ*_0_*H*=−69 mT contains a large transmission signal detected around 7.6 GHz. The oscillating behaviour indicates the propagation of spin waves through the YIG film between the two CPWs^20^,^21^. Their corresponding wavelength 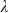 amounts to only 131±3 nm. At the crossing point (circle in Fig. 1d), the transmitted spin-wave signal is significantly enhanced by a factor of 6 compared with signals measured for non-degenerate eigenfrequencies of Py and YIG (blue arrows in Fig. 1d and Supplementary Fig. 2).

**Figure 2:**
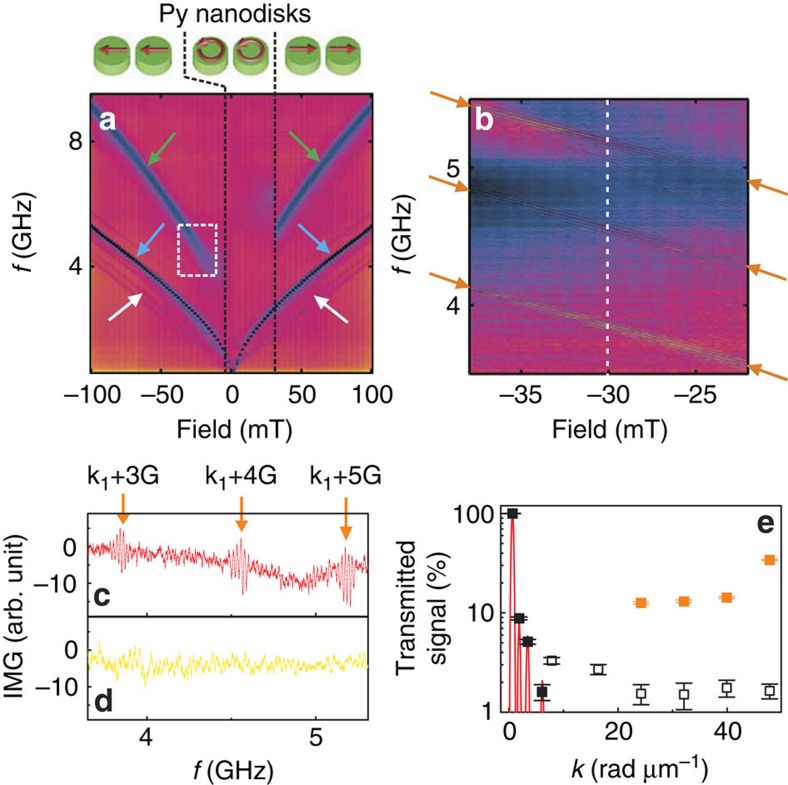
Reconfigurable microwave-to-magnon transducer exciting exchange-dominated spin waves. (**a**) Colour-coded absorption spectra *S*_11_ measured on the permalloy (Py)/YIG hybrid sample in reflection configuration. The field is varied from −100 to +100 mT. The blue arrows highlight excitation of YIG at *k*_1_. The green arrows highlight the FMR branch of Py nanodisks that show hysteretic behaviour. White arrows indicate spin-wave modes attributed to channelling effects due to the static stray field of the dots. (**b**) Colour-coded spectra *S*_12_ monitoring three sets of spin waves propagating through YIG (orange arrows). The parameter regime agrees with the white square in **a**. (**c**) Line spectrum *S*_12_ (imagninary (IMG) part) taken at −30 mT when nanodisks were saturated. Here, three resonantly enhanced modes are seen. (**d**) Line spectrum *S*_12_ taken at +30 mT when the nanodisks were in the unsaturated state. (**e**) Relative amplitudes of transmitted spin waves as a function of the wave vector *k* (experiment, symbols; expected values following ref. 20, red lines). Black, full (open) squares indicate modes *k*_1_–*k*_4_ (grating coupler modes without crossing of the nanodisk FMR branch) measured at −10 mT. Filled orange squares depict spin-wave modes in YIG with crossing of the nanodisk FMR branch. Error bars are a measure of the trace noise in *S*_12_ (as displayed in **d**) compared with the signal strength. All signals are normalized to the amplitude of the respective *k*_1_ mode taken as 100%.

In Fig. 2a, we show absorption spectra *S*_11_ taken in reflection configuration (selected lineplots are depicted in Supplementary Fig. 3). For *μ*_0_|*H*|>32 mT, all eigenfrequencies *f*(*H*) (dark) are mirrored with respect to *μ*_0_*H*=0 mT. The two pairs of most prominent branches (black) are highlighted by blue and green arrows. In case of the low-frequency one (blue arrow), *f*(*H*) follows roughly a square-root-like field dependence for *H*>0. The branch is consistent with *f*(*H*) of an unpatterned thin film of YIG when excited by a microstructured CPW^20^. It is noteworthy that the coercive field of the YIG film is only ∼0.5 mT (ref. 20). Its hysteretic behaviour is thus not resolved in Fig. 2a.

The branches marked by green arrows are at much higher frequency values and follow a different *f*(*H*) behaviour compared with YIG. These absorption features are in agreement with the FMR of the Py nanodisks that are magnetized along the in-plane magnetic field *H*. The nanodisks exhibit a large coercive field (switching field) of 32 mT and the hysteretic behaviour of the FMR frequencies is clearly observed. When *μ*_0_*H* is increased from −100 mT and reaches −6 mT, the upper prominent branch starts to vanish, indicating that the magnetization *M* of Py nanodisks turns away from the applied field orientation. The geometrical parameters of the nanodisks support the vortex state in the intermediate field regime^22^, as sketched in the inset. When the field is further increased to +33 mT, the nanodisks become saturated, with *M* parallel to *H*. For *μ*_0_|*H*|>33 mT, the upper prominent branch is hence present again. The black vertical lines in Fig. 2a highlight the three field regimes for different magnetic states of the nanodisks. The weak absorption lines (white arrow) at lower frequencies are attributed to channelling effects of spin waves in the thin YIG induced by the inhomogeneous stray fields of the nanodisks^9^. They will not be discussed here.

Figure 2b shows further colour-coded spectra *S*_12_ taken in the field and frequency regime marked by the white frame of Fig. 2a. Strikingly, we resolve three branches (highlighted by orange arrows). We note that the field dependence *f*(*H*) of each of the three branches agrees with *f*(*H*) of the YIG thin film and not with the steeper dependence attributed to the Py nanodisks. In Fig. 2c, we show the lineplot of the spectrum *S*_12_ taken at −30 mT. The observed oscillating signals are consistent with spin-wave excitation in YIG via the magnonic grating coupler effect^9^. The square lattice of saturated nanodisks provides us with reciprocal lattice vectors ±**G** that add to **k**_*i*_ of the CPW. The data of Fig. 2c are consistent with spin waves transmitted through YIG having wave vectors *k*_SW_=*k*_1_+3*G*, *k*_1_+4*G* and *k*_1_+5*G*, where 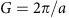. In the data of Fig. 2d taken at +30 mT we do not observe these transmission signals as will be discussed later.

We investigated the modes *k*_*i*_ and grating coupler modes *k*_1_+*nG* (*n*=1, 2, 3 …) over a broad magnetic field range. Following our analysis and comparison with calculated dispersion relations *f*(*k*) (Methods), we resolved spin waves with wave vectors ranging from *k*_1_ to *k*_4_ and from *k*_1_+1*G* to *k*_1_+6*G* in the Py/YIG hybrid sample. As the grating coupler modes followed the weaker field dependence of the YIG film, crossings with the steeper FMR branch of the Py nanodisks took place for large *k*_SW_. For each mode *k*_1_+*nG*, the crossing occurred at a different field value *H*_*n*_. In Fig. 2e, we summarize the signal strengths of *k*_*i*_ (filled black squares) and grating-coupler modes with (filled orange squares) and without (open squares) crossing the FMR branch. The red lines in Fig. 2e show the signal intensities as expected from the Fourier analysis of the field *h*_rf_ (compare with ref. 9). The first mode at *k*_1_=0.67 rad μm^−1^ (*λ*=9.4 μm ) is the most prominent one. The relative variation of the measured signal strengths taken at −10 mT matches quite well with the calculated variation in Fig. 2e. The open squares summarize the relative signal strengths of grating coupler modes *k*_1_+1*G* to *k*_1_+6*G* without provoking the crossing with the nanodisk FMR at −10 mT. For modes *k*_1_+3*G* to *k*_1_+6*G*, the crossing with the nanodisk FMR was possible. Strikingly, a considerably increased signal strength was found. The relevant fields *μ*_0_*H*_*n*_ (*n*=3…6) were −29, −42, −54 and −69 mT, respectively. For the wave vector of 48 rad μm^−1^ (*λ*=131 nm), we obtained a large signal strength of 34 % of mode *k*_1_. The measured group velocity of ∼0.86 km s^−1^ agrees rather well with the value calculated from the dispersion relation (Supplementary Fig. 4).

### Resonant grating couplers and sub-100 nm magnons

**Figure 3:**
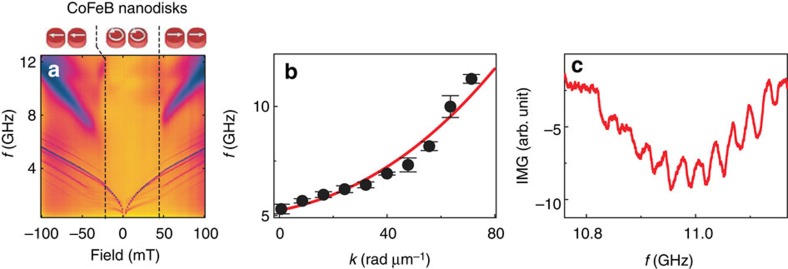
Spin waves with 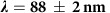 induced by resonating CoFeB nanodisks. (**a**) Colour-coded absorption spectra *S*_11_ measured on a CoFeB/YIG hybrid sample. The field is swept from −100 to +100 mT. Field regions for saturated and unsaturated states are highlighted by vertical lines. (**b**) Dispersion relation of the YIG thin film calculated for 90 mT (line). The black circles show eigenfrequencies obtained on CoFeB/YIG at the same absolute field. Error bars reflect the uncertainty of the resonance frequency due to noise and are extracted by taking the s.e. from fitting a Lorentz curve to the respective resonant peak in the measured spectra. (**c**) Transmission signal *S*_12_ that we attribute to *k*_SW_=*k*_1_+9*G*.

In the following, we present results on nanodisk arrays prepared from CoFeB, offering a larger FMR frequency *f*_0_ compared with Py. All other parameters are kept identical, such as the thickness, nanodisk diameter and lattice constant (Supplementary Table 1). Absorption spectra *S*_11_ of the CoFeB/YIG hybrid sample are colour coded in Fig. 3a. Owing to the large saturation magnetization *M*_S_ of CoFeB^21^, the field-dependent FMR frequencies for the saturated CoFeB nanodisks were higher by ∼3 GHz compared with Fig. 2a. This allowed us to induce crossings between the nanodisk FMR and spin waves in YIG at larger *k*_SW_ compared with Py. The observed spin-wave eigenfrequencies (symbols) and the calculated dispersion relation (line) are shown in Fig. 3b for *μ*_0_|*H*|=90 mT. The experimental data follow the predicted dependence *f*∝*k*^2^ caused by exchange interaction^23^. We extract a large relative signal strength of 38 % at *k*_SW_=*k*_1_+9*G*=71 rad μm^−1^ (Fig. 3c and Supplementary Fig. 5). The corresponding wavelength 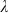 amounts to 88±2 nm. The propagation velocity is about 1.2 km s^−1^ for the *k*_1_+9*G* mode. Even larger velocities would be achieved by further increasing *k*_SW_ as *v*_g_ grows with increasing *k* (ref. 4). To further explore the grating coupler effect, we rotated an applied field of 8 mT in the film plane similar to that in ref. 20. In a specific angular regime (Supplementary Fig. 6), we observed weak transmission signals *S*_12_ around 12 GHz from which we estimated the so far shortest wavelength 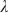 of 68±1 nm, that is, *k*=9.2 × 10^5^ rad cm^−1^. Here, the signal-to-noise ratio was close to 1 and too small to extract exact eigenfrequencies and group velocities. Still, these preliminary data substantiate the perspective of spin waves with *λ* of a few tens of nanometres excited by further optimized grating couplers.

### Reconfigurable microwave-to-magnon transducer

In Fig. 2c,d we compare two spectra *S*_12_ taken at the same absolute field *μ*_0_|*H*|, but two different states of the Py nanodisks. Strikingly, in the spectrum measured at +30 mT (Fig. 2d), we do not resolve the grating coupler modes. By means of different magnetic histories, we thus control the magnonic grating coupler effect between ON and OFF.

## Discussion

We now discuss the microscopic origin of the enhanced signal amplitudes. The CPW's microwave field **h**_rf_ coherently excites the FMR in the nanodisks at *f*=*f*_0_, leading to a large dynamic magnetic permeability^23^. Considering ref. 19, the dynamic stray field *h*_dip_ of the precessing spins locally enhances the microwave magnetic field and enlarges the torque acting on the magnetic moments in the YIG film near each nanodisk. As the nanodisks are thin platelets, the precessional motion is elliptical^23^ for which the in-plane dynamic magnetization components are larger compared with the out-of-plane components. This leads to surface charges mainly at their outer rim from where dynamic stray field lines originate. As nanodisks are positioned on the top surface of YIG, out-of-plane stray-field components are expected to be mainly relevant. Au *et al*.^19^ presented an analytical formula for *h*_dip_ of an individual cylindrical transducer. For a fixed sample design, the enhancement of *h*_rf_ via *h*_dip_ depended on materials properties according to 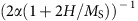 at *f*_0_; 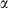 denoted the Gilbert damping parameter of the given transducer material. In contrast to ref. 19, our nanodisk arrays modulate spatially the enhancement effect. The spatial modulation leads to characteristic reciprocal lattice vectors and, in our case, to a resonantly enhanced grating coupler effect for the spin-wave excitation in YIG. The reciprocal lattice vectors add to *k*_*i*_ given by the bare CPW^9^. We attribute the slightly larger signal amplitude observed for CoFeB compared with Py (Supplementary Fig. 5) to the larger saturation magnetization *μ*_0_*M*_S_=1.8 T and the smaller damping parameter 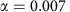 (ref. 21). Py shows *μ*_0_*M*_S_=1.0 T and 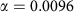 (ref. 24). To decrease 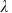 to the few 10-nm regime, the nanomagnets must provide an even larger resonance frequency than CoFeB. It is worth noting that a hexaferrite can exhibit an FMR eigenfrequency *f*_0_ of 53 GHz in zero magnetic field^25^. Exploiting such a material as a transducer, a spin-wave wavelength of ∼60 nm would be realized in YIG even without applying a magnetic field. The frequency *f*_0_ of hexaferrite can be further increased by *H*>0. These features would allow one to observe branch crossings beyond *n*=9. Furthermore, state-of-the-art nanolithography would allow to decrease the lattice constant *a* by about a factor of 10 compared with our prototype devices. The corresponding reciprocal lattice vectors *G* can thus be made one order of magnitude larger, suggesting wavelengths 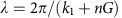 on the 10-nm length scale for *n*=6.

We assume the resonantly enhanced spin-wave amplitude to be even larger than given by the value of, for example, 38% stated for the CoFeB/YIG hybrid sample. This value is extracted from the inductive voltage picked up by the detector CPW and normalized to the amplitude at *k*_1_. At *k*_1_, the spin-precessional motion induces the maximum voltage signal in the CPW as the spin-precessional motion can be in phase across the full width of the signal line. This large signal is illustrated by the left-most peak (red line) in Fig. 2e. Spin waves with wave vectors *k*>*k*_1_ induce much smaller voltage signals, as out-of-phase components underneath the signal line compensate each other at all times. For specific (commensurable) wavelengths, the induced signal is zero, such that a given CPW would not detect the corresponding spin wave at all. For the grating coupler mode with *n*=9, the wavelength is extremely small compared with the CPW's signal line width and even smaller than the diameter of the nanodisks. The spin wave cannot uniformly excite a nanodisk, leading to a small inductive response in the CPW. For large *n*, we therefore expect the absolute spin-wave signal in YIG to be larger than evaluated from the width-averaged voltage induced in the CPW. To measure the absolute spin-precessional amplitude a dynamic technique with high spatial resolution would be needed, such as synchrotron-based X-ray magnetic circular dichroism. Following ref. 19, resonant microwave-to-magnon transducers should be as close as possible to magnonic devices as spacer layers degrade the excitation efficiency (compare Fig. 1e in ref. 19).

For the present study, we decided for a lattice constant of 800 nm and avoided to bring nanodisks too close together. In case of smaller separation, strongly coupled nanodisks are expected. An artificial band structure might be formed such that forbidden frequency gaps would open. Frequency gaps would counteract efficient spin-wave transmission over broad frequency and wave vector regimes. Still, studies on coupled dots would be very interesting in terms of excitation strength and damping.

Our CPW-based technique is versatile for both fundamental and applied research. A VNA provides a very high frequency resolution (about 1 Hz at 10 GHz), which is better compared with inelastic light scattering, neutron scattering, scanning tunnelling microscopy or electron loss spectroscopy at room temperature. We have focussed on the Damon–Eshbach configuration where *k*_SW_ is perpendicular to *H*. Other configurations work in a similar way. The excellent frequency resolution of the VNA combined with the resonant grating coupler effect demonstrated here thus opens a new horizon for exploration and functionalization of exchange-dominated spin waves. For example, one might excite coherent short-wavelength spin waves in different inputs of multi-terminal magnonic devices such as the magnon transistors presented in ref. 26. The relevant coherency can be induced by either a CPW common to the different inputs or a microwave cavity.

Importantly, the group velocity scales with wave vector 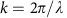 in the first third of the first Brillouin zone^4^. For YIG, that is, the key material in magnonics offering the smallest propagation losses, signal speeds of a few km s^−1^ would be realistic. These velocities are comparable to surface acoustic waves (SAWs) that are currently used in microwave technology. SAW-based microwave electronics such as delay lines, filters and duplexers are building blocks of billions of cell phones. Exchange-dominated spin waves as discussed here offer a wavelength that is two orders of magnitude smaller compared with SAWs. Magnonics thus allows for unprecedented miniaturization of microwave components. This aspect is also interesting for the internet of things consisting of billions of smart devices communicating in a wireless manner^11^.

The reconfigurable characteristics of Fig. 2c,d contribute to a topic of great current interest and provide additional functionality^11^,^27^,^28^. Reconfiguration may be achieved by an applied magnetic field as used here or by microwave-assisted switching. Microwave-assisted switching has already been demonstrated in mesoscopic magnets with and without vortex states^29^,^30^. Consequently, tailored microwave pulses would allow one to reconfigure nanodisks under a preselected antenna. This offers further possibilities in grating coupler-based microwave circuits. The constructive superposition of two microwave signals sent to the same CPW could be used to induce the switching of nanodisks. The superimposed signal could erase the grating coupler effect and the relevant microwave signal would not be transmitted to the detector CPW. This operation would mimic an Exclusive OR (XOR) logic gate functionality. Importantly, our experiments show that six periods of nanodisks between the ground lines of a CPW are already sufficient to induce a grating couler effect. This finding is important for the miniaturization and high-density integration of spin-wave transducers with microwave circuitry on a chip. In two dimensions, five Bravais lattices of different lattice symmetries are possible for microwave-to-spin-wave transduction at optimized magnonic properties. It is noteworthy that from small array to small array, different lateral or materials parameters could be used to implement different operational frequencies and wavelengths on the same chip if required. Beyond that, we expect grating couplers with selectively switched nanodisks to allow for the generation of complex wave patterns on a chip.

In ref. 23, it was discussed for a YIG sphere that magnon–magnon scattering processes were found to increase the linewidth 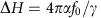, measured at *k*=0, with increasing wave vector *k* (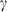 is the gyromagnetic ratio). For our largest wave vector of 9.2 × 10^5^ rad cm^−1^, four-magnon scattering is expected to contribute most^23^. Figure 11.6 of ref. 23 suggests the linewidth measured at *k*=0 to increase by ∼0.9 Oe at *k*=9.2 × 10^5^ rad cm^−1^ and 35 GHz. Assuming a spin wave with 9.2 × 10^5^ rad cm^−1^ to be operated at 35 GHz for *μ*_0_*H*=50 mT, the relevant relaxation time 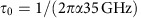 without four-magnon scattering amounts to ≈20 ns. From the calculated spin-wave dispersion relation *f*(*k*), we extract a group velocity 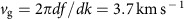 at *k*=9.2 × 10^5^ rad cm^−1^. The decay length *l*_d_ given by 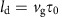 is then estimated to be around 73 μm, without additional four-magnon scattering. Considering the linewidth of ∼3 Oe at *k*=0 for our thin-film YIG^31^, the additional wave-vector-dependent damping of 0.9 Oe is expected to increase Δ*H* by a factor of 1.3. Assuming a linear relationship between Δ*H* and 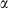 at a given frequency, we expect the decay length to be reduced by a corresponding amount of ∼30%, leading to an estimated value *l*_d_ of 50 μm. This value outrivals the typical mean free path of electrons by three orders of magnitude. Hence, the key advantage of magnonics in information technology, that is, the coherent and parallel processing of information within large cellular networks^12^, can now be realized.

In conclusion, we explored the spin-wave excitation using magnetic nanoresonators integrated to conventional microwave antenna. We realized reconfigurable microwave-to-spin-wave transducers at GHz frequencies that permitted us exciting and detecting exchange-dominated spin waves. In our non-optimized devices, the minimum wavelength for transmitted spin waves was clearly below 100 nm. Further reduction is straightforwardly possible. Thereby, wavelengths become accessible in solid-state devices that are known from soft X-rays. We are convinced that our results boost the integration of nanomagnonics with microwave circuits and allow to harvest the rich advantages of spin-wave-based applications.

## Methods

### Sample fabrication

We grew thin YIG films on Gadolinium Gallium Garnet (111) substrates using pulsed laser deposition. The growth temperature was set to 650 °C and oxygen pressure was 0.25 mbar. The YIG thickness was measured using X-ray reflectometry. Root-mean-square roughness was better than 0.5 nm. The Gilbert damping parameter 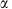 was evaluated to be 2.3 × 10^−4^. We used electron beam lithography and Ar ion milling to form mesas with 45° edges to avoid reflection of the spin waves. The width of the mesa was 300 μm. Using electron beam lithography and lift-off processing, the nanodisks were prepared from either evaporated polycrystalline Ni_80_Fe_20_ or magnetron-sputtered amorphous CoFeB. The composition of the target was Co_20_Fe_60_B_20_. We did not apply a cleaning process for the interface between YIG and the nanodisks. Using again electron beam lithography and lift-off processing, CPWs were integrated on the YIG mesa and nanodisks to perform broadband spin-wave spectroscopy. These CPWs were from Au and Cr, and were designed having 2.1-μm-wide signal lines and ground lines, and 1.4-μm-wide gaps between them. The distance *s* between two central conductors of adjacent CPWs was 30 μm. Supplementary Fig. 1 displays a microscopy image of one of the CPWs prepared on top of, both, a Py nanodisk array and the ferrimagnetic thin-film insulator YIG. We prepared the nanodisks on the top surface of the YIG film. Intentionally, we did not embed them, to allow for spin-wave propagation through the unpatterned YIG film. The arrays did not exist between neighbouring CPWs. We stress that the specific relation between precessional frequencies of nanodisks (FMR) and film (spin waves) was intentionally opposite to ref. 9. In ref. 9 the grating coupler effect was reported for all-metal-based magnetic devices. Owing to the reversed relation, we were able to force the field-dependent FMR of the nanodisks to be degenerate with short-wavelength spin-wave eigenfrequencies in YIG. Such a resonant grating coupler was not realized in ref. 9.

### Propagating spin-wave spectroscopy

To measure spin-wave propagation between two CPWs via scattering parameter *S*_12_, the CPWs were connected to a VNA that provided a microwave current at GHz frequencies *f* to one of the CPWs. Through 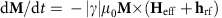, the accompanying magnetic field **h**_rf_ excited the magnetic moments underneath the antenna^32^,^33^. The microwave power was set at −5 dBm (316 μW) or smaller. As the saturated nanodisks exhibited both different effective fields **H**_eff_ (due to shape anisotropy)^23^ and larger magnetization values *M* compared with YIG, their FMR was at larger frequency *f* compared with the uniform precessional motion of the thin YIG film, that is, the spin excitation with wave vector *k*_SW_=0. The inhomogeneous magnetic field around a CPW excited the spins at a distinct set of wave vectors **k**_**i**_ with *i*=1, 2, 3, … as was shown in ref. 20. The distribution of wave vectors *k* provided by a microwave antenna was calculated from Fourier transformation using the lateral dimensions, that is, signal line width, ground line width and their spacing^34^. Wave vectors **k**_**i**_ generated by the CPW were perpendicular to and in the plane of the CPW. The absolute amplitude *S*_12_ measured at *k*_1_ depended on *H* and decreased with increasing *H*. The wavelengths of spin waves *i* excited directly be the CPW were calculated according to 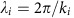 with *k*_*i*_ extracted from the Fourier analysis of the microwave field. The wavelengths 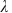 of grating-coupler-induced spin waves were determined via the calculated spin-wave dispersion relations 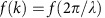 (see main text) that allowed us to relate the given VNA frequency *f* to the relevant wave vector 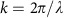. The error bar for 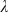 was taken from the linewidth *δf* of the envelope function and extracted from the slope of the dispersion relation *f*(*k*) around the corresponding wave vector *k*. We attributed the observation of the FMR branch of the Py nanodisks in the *S*_12_ data of Fig. 1c (black) of the main text to the remaining electromagnetic cross-talk that existed between the two CPWs being separated by 30 μm. In Supplementary Fig. 2a, the eigenfrequencies of the two branches are summarized that were highlighted by orange and green arrows in Fig. 1c of the main text. Eigenfrequencies were extracted from transmission data displaying oscillating signals as shown in Fig. 1 of the main text in that we defined envelope functions connecting either the local maxima or minima. The eigenfrequency was taken at the frequency where the two envelope functions exhibited the largest difference. In Supplementary Fig. 2b we show the field dependence of the peak-to-peak amplitude in *S*_12_ when spin waves propagate through the YIG film (orange). Clearly, the amplitude is significantly enhanced at the crossing point of the two branches. Without the crossing, the spin-wave amplitude is always at a small signal level (Supplementary Fig. 2c,d) as was reported in ref. 9. Supplementary Table 1 summarizes wavelengths of spin waves observed in different samples with and without nanomagnet arrays for comparison. The grating coupler effect allowed us to increase the wave vector *k*_1_ by adding multiples of the reciprocal lattice vector 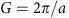 (ref. 9), where *a* amounted to 800 nm.

### Group velocity calculation

The oscillation feature of propagating spin-wave signal^33^ provided us with the group velocity 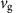 of the respective spin-wave mode. As studied in refs 21, 35, the frequency separation Δ*f* (defined in Fig. 1e of the main text) reflects a change in the phase by 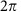, which is accumulated by spin waves propagating along the distance *s*=30 μm. The group velocity 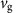 is calculated from Δ*f* according to ref. 21


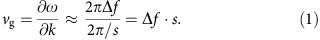


where *s* is the distance between two CPW signal lines as indicated in Fig. 1a of the main text.

### Absorption spectra

Supplementary Fig. 3a,b shows the reflection spin-wave spectra *S*_11_ (absorption) measured using one-and-the-same antenna for excitation and detection. In the absorption spectrum at −69 mT, when the crossing occurs, we observe two peaks separated by ∼150 MHz (highlighted by arrows) and attribute them to the two grating coupler modes *k*_1_±6*G*. In the transmission spectra, only one of them (*k*_1_+6*G*) is prominent. The wave vector *k*_1_+6*G* was determined by comparison with the calculated spin-wave dispersion relation (see below).

### Analysis of dispersion relations and group velocities

In our experiment the field **H** was applied perpendicular to the spin-wave vector *k*_SW_. Thus, we addressed the so-called Damon–Eshbach mode configuration. Following refs 20, 36, we calculated the relevant dispersion relation *f*(*k*) in YIG (solid line in Supplementary Fig. 4a) using a thickness of 20 nm, the magnetization *μ*_0_*M*_S_=0.176 T, an exchange constant 0.192 × 10^−6^ erg cm^−1^ and *μ*_0_|*H*|=69 mT. In Supplementary Fig. 4a, we plot the extracted eigenfrequencies (symbols) as a function of the anticipated wave vector *k*. The experimental data taken on the Py/YIG hybrid sample follow quite well the calculated dispersion relation substantiating the mode allocation. In the literature, exchange constants up to ∼0.4 × 10^−6^ erg cm^−1^ were reported for (thin) YIG. Using such a large value we remodelled well the spin-wave eigenfrequencies with the shortest wavelength in the CoFeB/YIG hybrid sample, but not the intermediate wavelengths and not the results obtained on the Py/YIG hybrid sample. Calculated spin-wave group velocities of all the different modes from *k*_1_ up to *k*_1_+6*G* observed on the Py/YIG hybrid sample are shown in Supplementary Fig. 4b. It is noteworthy that 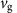 increases with *k*, consistent with the theoretical prediction (line). This behaviour is opposite to the long-wavelength regime reported for bare YIG in ref. 20 and underlines that we generated exchange-dominated spin waves in the thin-film YIG. We extracted 0.86 km s^−1^ for the spin wave with 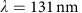 (mode *k*_1_+6*G*). This value is larger by a factor of two compared with mode *k*_1_ directly excited by the CPW. For the resonantly enhanced spin wave with 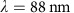 in the CoFeB/YIG hybrid sample, the spin-wave velocity was measured to be 1.2 km s^−1^ and hence three times larger compared with the long-wavelength limit at the same applied magnetic field *H*.

### Spin-wave amplitudes by resonant grating couplers

The microwave current in the CPW is in one direction in the signal line and the opposite direction in the two ground lines. This corresponding phase relation in the CPW's magnetic microwave field *h*_rf_ creates the finite wave vectors *k*_*i*_ (*i*=1, 2, 3,..) for the spin waves and an excitation efficiency as displayed by the red curve in Fig. 2e of the main text. The red curve represents the outcome of the Fourier transformation of the current distribution described above. The measured signal strengths do follow the expected excitation efficiency for the spin waves with *k*_1_ to *k*_4_ directly excited by the bare CPW. For *k* being larger than *k*_4_, the grating coupler effect comes into play and rules the excitation efficiency—the measured signal strengths do not follow the outcome of the Fourier transformation any longer.

In Supplementary Fig. 6 we show angular-dependent transmission signals from which we extracted the shortest spin-wave wavelength of 68 nm. At the small field of 8 mT that we rotated, the grating coupler effect was nonresonant. The corresponding spin-wave signal was weak and close to a signal-to-noise ratio of 1:1. A detailed inspection and high-resolution computer screens were used to identify angular-dependent branches. Circles highlight two of the regions where branches were suggested. The signal was too small to extract a group velocity from a contrast oscillation. From such weak angular-dependent branches compared with calculated dispersion relations, we concluded that our non-optimized nanomagnet array, prepared via conventional nanolithography, induced spin waves with a minimum wavelength of 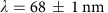.

## Additional information

**How to cite this article:** Yu, H. *et al*. Approaching soft X-ray wavelengths in nanomagnet-based microwave technology. *Nat. Commun.* 7:11255 doi: 10.1038/ncomms11255 (2016).

## Supplementary Material

Supplementary Information Supplementary Figures 1-6 and Supplementary Table 1
