# Supplementary material for: Approaching soft X-ray wavelengths in nanomagnet-based microwave technology
Source: Nat Commun. 2016 Apr 11;7:11255. doi: 10.1038/ncomms11255 (PMC4831022; doi:10.1038/ncomms11255)
Supplement: Supplementary Information — Supplementary Figures 1-6 and Supplementary Table 1 [file ncomms11255-s1.pdf]

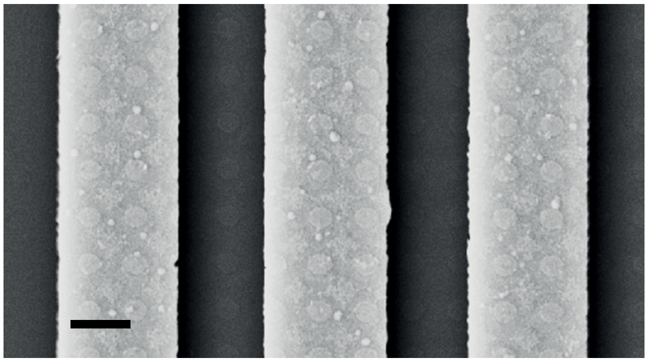

Supplementary Figure 1. **Scanning electron microscopy.** A coplanar waveguide (bright) consisting of a center signal line and two ground lines. A permalloy nanodisk array was prepared on top of yttrium iron garnet. The nanodisks underneath the metallic leads of the coplanar waveguide (CPW) provide the largest contrast. There are six columns (periods) of nanodisks between the two inner edges of the ground lines of the CPW. The nanodisk array extends along the length of the CPW. It does not exist between two neighboring CPWs whose center-to-center distance of the signal lines amounts to 30 micrometers. The scale bar represents 1000 nm.

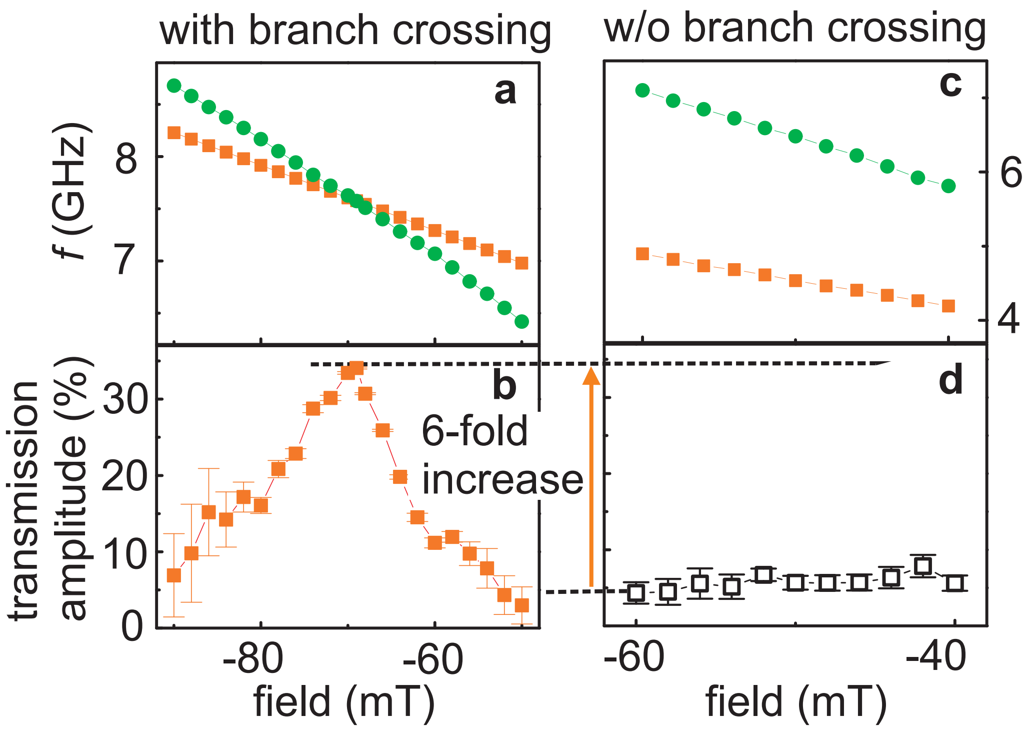

Supplementary Figure 2. **Spin waves with $\lambda = 131 \pm 3$ nm in yttrium iron garnet using permalloy nanodisks.** (**a**) Field-dependent eigenfrequencies of excitations in yttrium iron garnet (squares) and permalloy nanodisks (circles). (**b**) Peak-to-peak amplitude detected in S_{12} normalized to the signal strength at k_1. The signal takes its maximum value when the branches cross in graph **a**. (**c**) Field-dependent eigenfrequencies of excitations in yttrium iron garnet (squares) and permalloy nanodisks (circles) outside a branch-crossing region. (**d**) Peak-to-peak amplitude of the corresponding spin-wave signal. The error bars in (b) and (d) are extracted from the trace noise of the spectra S_{12} at the respective applied fields.

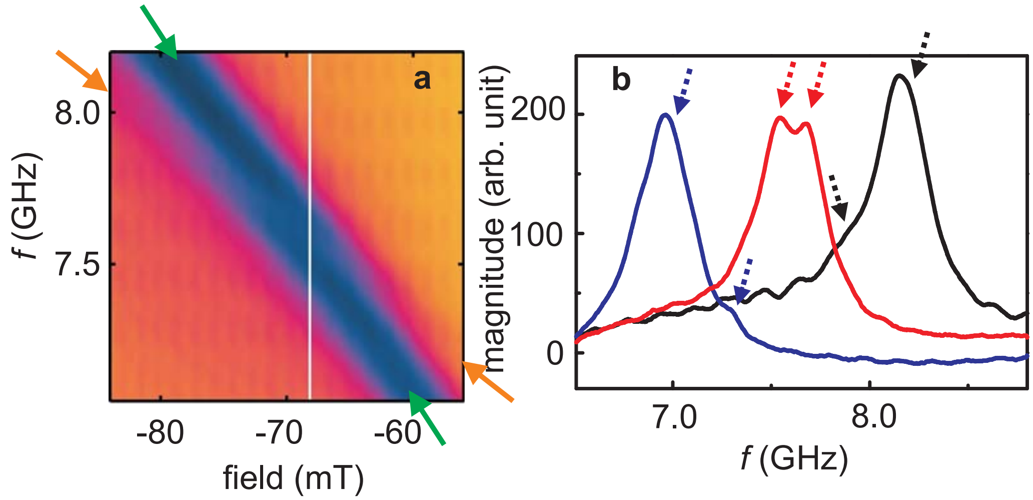

Supplementary Figure 3. Reflection S_{11} measured on yttrium iron garnet containing permalloy nanodisks. (**a**) Color-coded reflection data S_{11} measured on yttrium iron garnet with a permalloy nanodisk array underneath the coplanar waveguide. Black (orange) color indicates a large (zero) signal (compare linescans in **b**). At the white line a spectrum is missing due to an error while taking the data. Orange arrows indicate a mode in yttrium iron garnet and green ones indicate the mode of permalloy nanodisks. (**b**) Line spectra extracted at the field values of -80 mT (black), -69 mT (red), -60 mT (blue). For spectra at -80 and -60 mT the two arrows indicate the two resonances attributed to the nanodisk array and yttrium iron garnet. At -69 mT, when the crossing occurs, we attribute the two peaks (separated by about 150 MHz and highlighted by arrows) to the two grating coupler modes $k_1 \pm 6G$.

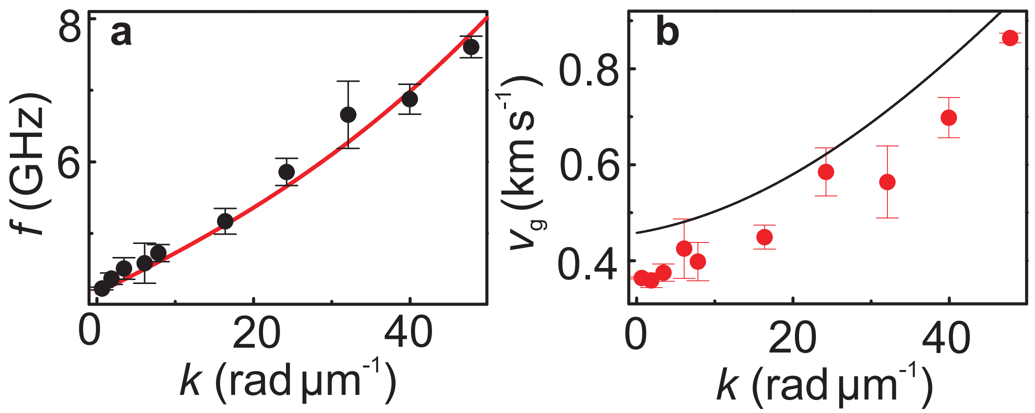

Supplementary Figure 4. **Dispersion relations and calculated group velocities.** (**a**) Dispersion relation (line) calculated for the DE mode in a 20 nm thick yttrium iron garnet film at 69 mT. Black circles with error bars represent measured spin-wave frequencies attributed to modes k_1 to k_4 and $k_1 + 1G$ to $k_1 + 6G$ at the same absolute field. Error bars reflect the standard error when fitting a Lorentz curve to the corresponding resonance in S_{11}. (**b**) Group velocities as a function of k measured at 69 mT. The line indicates the theoretically expected values. The data are for spin waves in the permalloy/yttrium iron garnet hybrid sample. The error bar reflects the statistical variation when reading out Δf from individual oscillations for the relevant k.

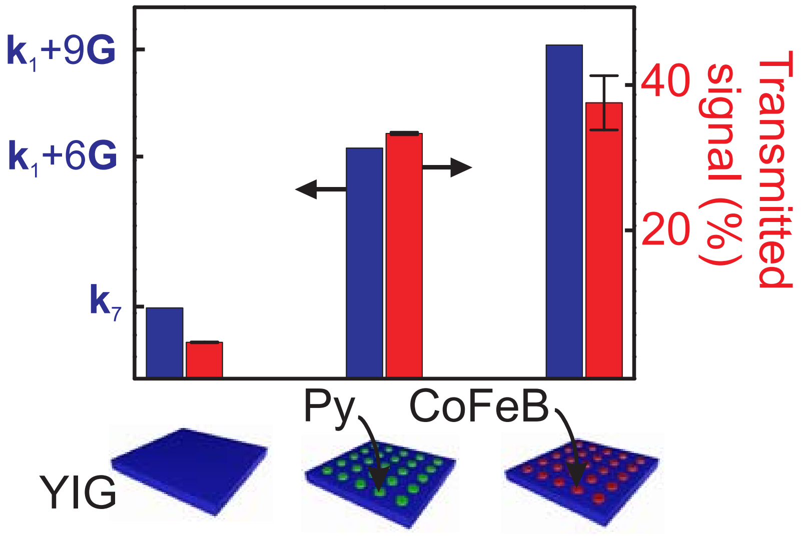

Supplementary Figure 5. **Comparison between three different samples.** Blue pillars indicate the largest k vector observed in a given sample. Red pillars state the signal amplitude for the largest k normalized to the respective k_1 mode given in %. From left to right, we compare a bare YIG film with the permalloy/yttrium iron garnet and CoFeB/yttrium iron garnet hybrid samples, respectively. For CoFeB/yttrium iron garnet we find the largest wave vectors and signal amplitudes. Error bars are a measure of the trace noise compared to the signal strength.

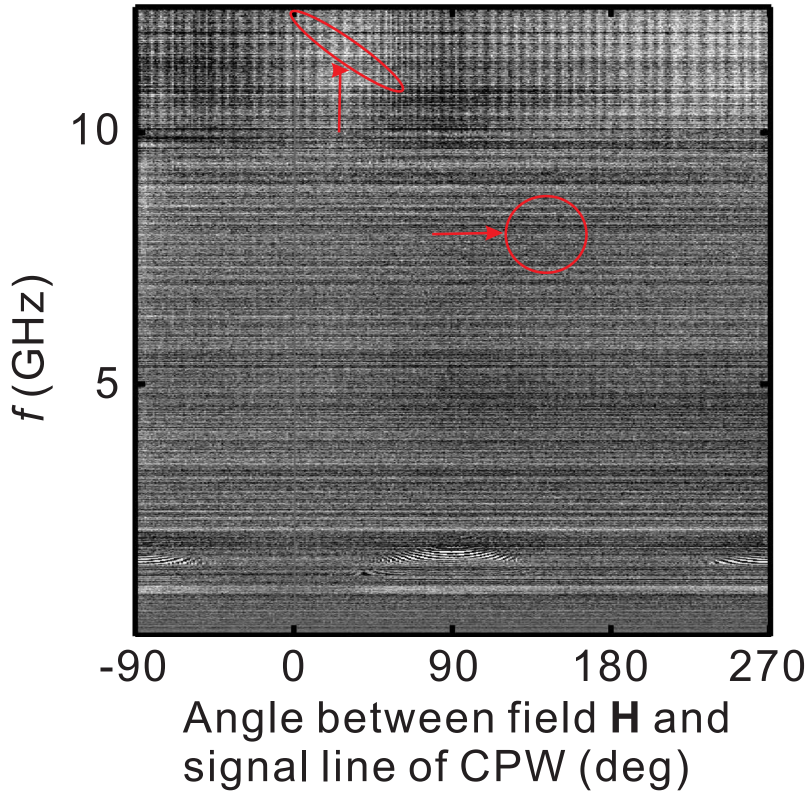

Supplementary Figure 6. **Angular dependent S_{12} measured on yttrium iron garnet with CoFeB nanodisks.** The color-coded data summarize transmission signals measured between two coplanar waveguides (CPWs) when a field $\mu_0 H$ of 8 mT was rotated in the plane of the yttrium iron garnet thin film. As $\mathbf{M}$ of yttrium iron garnet follows the orientation of $\mathbf{H}$, a transmitted spin wave $k_1 \pm nG$ varies characteristically its eigenfrequency. The vertical arrow and circle indicate a region where an angular-dependent branch with signal-to-noise ratio close to one suggests a transmitted spin wave with λ of about 68 nm considering the relevant spin-wave dispersion relations. The horizontal arrow and circle indicate a region where a detailed inspection suggests branches of spin waves with opposing angular dependence that cross each other. At low frequency, prominent transmission signals are see (black-white-black oscillating contrast). Strictly vertical and strictly horizontal stripe-like features are due to the coplanar waveguides and data analysis in that we removed slowly varying backgrounds from spectra taken at successive angles.

	Film thickness t_1	Period a	Nanodisk thickness t_2	Embedded depth t_3	Largest multiple of G observed	Spin-wave wavelength
CoFeB/YIG with branch crossing	20 nm	800	15	0	$9G$	68 nm
Py/YIG with branch crossing	20 nm	800	15	0	$6G$	131 nm
Py/CoFeB no crossing possible	48 nm	800	25	15	$1G$	716 nm
YIG without grating	20 nm	N/A	N/A	N/A	$0G$	9.4 μm

Supplementary Table I. **Samples and magnetic nanoresonators.** Comparison of sample parameters, materials, i.e., permalloy (Py), yttrium iron garnet (YIG) and CoFeB, and minimum spin-wave wavelengths observed in magnetic thin films with and without the branch crossing allowed by the degenerate ferromagnetic resonance of a nanodisk grating. We integrated coplanar waveguides of the same design providing $\lambda_1 = 2\pi/k_1 = 9.4$ μm.
